# Inpatient Trauma Mortality after Implementation of the Affordable Care Act in Illinois

**DOI:** 10.5811/westjem.2017.10.34949

**Published:** 2018-02-19

**Authors:** Paul L. Weygandt, Scott M. Dresden, Emilie S. Powell, Joe Feinglass

**Affiliations:** * Northwestern University Feinberg School of Medicine, Department of Emergency Medicine, Chicago, Illinois; † Northwestern University Feinberg School of Medicine, Division of General Internal Medicine and Geriatrics, Chicago, Illinois

## Abstract

**Introduction:**

Illinois hospitals have experienced a marked decrease in the number of uninsured patients after implementation of the Affordable Care Act (ACA). However, the full impact of health insurance expansion on trauma mortality is still unknown. The objective of this study was to determine the impact of ACA insurance expansion on trauma patients hospitalized in Illinois.

**Methods:**

We performed a retrospective cohort study of 87,001 trauma inpatients from third quarter 2010 through second quarter 2015, which spans the implementation of the ACA in Illinois. We examined the effects of insurance expansion on trauma mortality using multivariable Poisson regression.

**Results:**

There was no significant difference in mortality comparing the post-ACA period to the pre-ACA period incident rate ratio (IRR)=1.05 (95% confidence interval [CI] [0.93–1.17]). However, mortality was significantly higher among the uninsured in the post-ACA period when compared with the pre-ACA uninsured population IRR=1.46 (95% CI [1.14–1.88]).

**Conclusion:**

While the ACA has reduced the number of uninsured trauma patients in Illinois, we found no significant decrease in inpatient trauma mortality. However, the group that remains uninsured after ACA implementation appears to be particularly vulnerable. This group should be studied in order to reduce disparate outcomes after trauma.

## INTRODUCTION

Disparities in health outcomes between insured and uninsured patients have been demonstrated throughout medicine^1^ and in various surgical settings including trauma^2^ and emergency surgery.^3^ Insurance-related disparities in trauma outcomes are unique, given that the Emergency Medical Treatment and Active Labor Act (EMTALA) “ensures public access to emergency services regardless of ability to pay.”^4^

Recent studies have shown insurance-related disparities in outcomes after both blunt and penetrating trauma,^5^,^6^ with consistently increased mortality for uninsured patients.^2^ Despite a growing body of literature demonstrating disparities in trauma outcomes on the basis of race and socioeconomic status, causal mechanisms are poorly understood.^2^ Factors that may contribute to observed disparities include host factors such as undiagnosed comorbid disease,^7^ prehospital factors such as transport time and trauma catchment areas,^8^,^9^ hospital and provider factors such as differential hospital performance and provider implicit bias,^10^,^11^ and access to post-hospital and rehabilitation care.^2^,^12^

While the causal mechanisms of worsened outcomes for uninsured patients after trauma remain unclear, we saw Affordable Care Act (ACA)-related healthcare expansion as a natural experiment. The ACA was designed in part to provide access to care for many of the nation’s uninsured; given increased mortality for the uninsured following trauma, we expected a concomitant decrease in mortality. Patients may have experienced improved outcomes after enactment of the ACA due to greater access to higher quality hospitals, improved inpatient care, or improved rehabilitation care in the hospital or beyond.

While the ACA provides a potential opportunity to reduce disparities in care and outcomes, results have thus far been mixed.^13^ A recent study surprisingly suggested an increase in trauma mortality after Massachusetts healthcare reform.^14^ It is unclear if this effect will be seen nationwide, especially in light of the bulk of previous literature showing poorer outcomes for uninsured trauma patients when compared to their insured counterparts.^2^ In Illinois, the ACA led to a 24% decrease in the uninsured population, though its impact on insurance-related trauma disparities has yet to be determined.^15^ The primary aim of this study was to determine the impact of ACA-related insurance expansion on trauma mortality in Illinois. The secondary aim was to determine the remaining insurance-level effects of the ACA on trauma mortality in Illinois.

## METHODS

### Study Design

This was a retrospective, cohort study of Illinois trauma patients. Because we looked at publicly available data that were de-identified, the study was determined to be exempt by the Northwestern University Institutional Review Board.

### Study Setting and Population

We performed a retrospective cohort study of administrative data from the Illinois Hospital Association Health Care and Hospital Data Reporting Services (COMPdata) matched to the American Community Survey (ACS) by zip code from third quarter 2010 through second quarter 2015. Cases were defined as patients aged 18–64 who were admitted as inpatients through the emergency department (ED) with trauma-related diagnoses.

### Study Protocol

We identified trauma-related *International Classification of Diseases, Ninth Revision* (*ICD-9*) codes (800–959.9) and an ECODE related to major mechanisms of trauma (cut/pierce, fall, gunshot wound, self-inflicted gunshot wound, motor vehicle collision, or other blunt injuries). Other blunt injuries included other traffic accidents and horseback-rider injuries; however, we excluded aviation and boating injuries (E930–940). Admissions were excluded if they had only *ICD-9* codes related to foreign bodies (930–939), burns (940–949), or complications of trauma (958) as has been done in prior studies.^14^ We matched records from the COMPdata dataset by patient zip code to five-year (2009–2013) ACS estimates of median household income.

To adjust for severity of injuries, we employed the Trauma Mortality Prediction Model (TMPM), which calculates the individual probability of mortality based on the extent of injury as defined by *ICD-9* injury codes.^16^ We excluded admissions if they lacked sufficient injury data for the TMPM to assign an associated probability of mortality. We calculated the Charlson Comorbidity Index (CCI) for each patient to assess preexisting comorbidities and categorized our patients as not ill (CCI=0), moderately ill (CCI>0 and <3), and severely ill (CCI>=3).^17^ We dichotomized admissions as pre-ACA (2010–2013) vs. post-ACA (2014–2015) and tested the significance of the association of post-ACA period and mortality.

**Population Health Research Capsule**

What do we already know about this issue?

Uninsured patients die at higher rates after trauma; however, it remains unclear if Affordable Care Act (ACA)-related healthcare expansion will impact trauma mortality.

What was the research question?

Did ACA-related healthcare expansion in Illinois reduce trauma mortality?

What was the major finding of the study?

The ACA did not reduce trauma mortality over the study period, and insurance-related disparities persisted.

How does this improve population health?

Healthcare coverage alone is not sufficient to reduce trauma mortality or insurance-related disparities, highlighting the need for future studies and interventions.

### Outcome Measures

Our primary outcome of interest was the adjusted incident rate ratio (IRR) for trauma mortality comparing post-ACA trauma patients to pre-ACA trauma patients. Our secondary outcome of interest was the adjusted IRR for trauma mortality comparing post-ACA trauma patients to pre-ACA trauma population among the uninsured.

### Statistical Analysis

Within our dataset we identified variables that have been demonstrated in the literature to be predictive of trauma mortality and assessed those variables with standard univariate statistics. We subsequently performed two-tailed t-tests for proportions and chi-square tests to identify those variables associated with mortality. Given the low incidence of mortality in this dataset, we used multivariable Poisson regression to provide IRRs that approximate relative risk.^18^

The Poisson regression models included key sociodemographic and clinical variables shown in prior studies to be independent predictors of trauma-related mortality, including age,^19^,^20^ sex,^21^ race,^22^ socioeconomic status,^23^,^24^ mechanism of injury,^25^ injury severity,^16^,^26^,^27^ and shock as defined by systolic blood pressure less than 90 mm Hg.^28^ Our final model included patient age category (18–25, 26–33, 34–45, 46–55, 55–64), sex, race, residence in a low-income zip code (<$35,000 median household income), quarter (to adjust for seasonal variation), insurance status (Medicaid, Medicare, uninsured, private), mechanism of injury, injury severity, comorbidities, shock, and hospital trauma volume as quartiles of total ED trauma visits over the study period. We performed statistical analyses using Stata version 12.1, College Station, TX.^29^

## RESULTS

**Figure 1:**
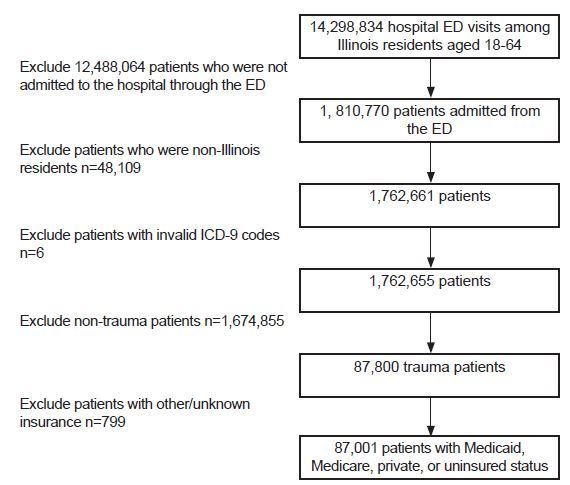
Flowchart for inpatient trauma dataset. *ED*, emergency department; *ICD-9*, International Classification of Diseases, Ninth Revision.

Over the study period, there were 14,298,834 hospital ED visits among Illinois residents age 18–64. We excluded 12,488,064 patients who were not admitted through the ED (discharges, transfers, and patients who were dead on arrival); 1,810,770 patients were admitted through the ED. We excluded 48,109 patients who were not Illinois residents, six with invalid *ICD-9* codes, 1,674,855 who were not trauma patients, and 799 patients with unknown or other insurance types. A total of 87,001 trauma patients met inclusion criteria (Figure 1). Characteristics of this cohort can be seen in Table 1.

Median age was 47, and 66% of the patients were male. Fifty-nine percent of patients were White, 21% were Black, 13% were Latino, and 7% were of other minority or unknown race. Overall, 13% lived in low-income zip codes. Most of the patients in this cohort suffered from falls, MVCs, or other blunt injuries. Penetrating trauma comprised approximately 11% of injuries, including gunshot wounds (GSWs) that accounted for 5.8%. One percent had shock. Most patients were not ill or moderately ill, while only 6% of the trauma population was severely ill at the time of their ED admission. Median probability of mortality based on injury severity as assessed by the TMPM was 1.3%, while approximately 1.7% of our cohort of hospitalized trauma patients died. GSW victims comprised only 5.8% of patients in our study population, but accounted for 18% of deaths overall and 32% of deaths among the uninsured (data not shown).

**Figure 2:**
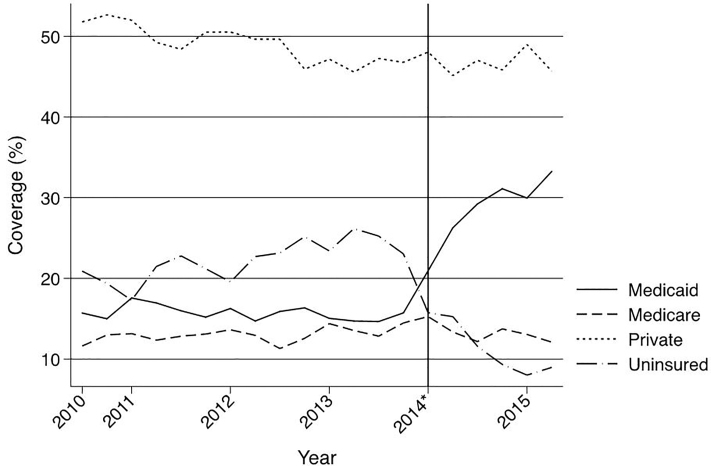
Primary payer mix for trauma inpatients in Illinois from third quarter 2010 through second quarter 2015. *Indicates January 1, 2014, which marks the beginning of insurance expansion in Illinois.

Within our cohort of trauma patients, the number of uninsured dropped 11% after the implementation of the ACA (95% confidence interval [CI] [10.24–11.26]). The number of Medicaid patients increased by 13% (95% CI [12.23–13.48]), and there was no significant change in the number of privately insured patients (−2.4% 95% CI [−3.15–1.59]) (Figure 2).

**Figure 3:**
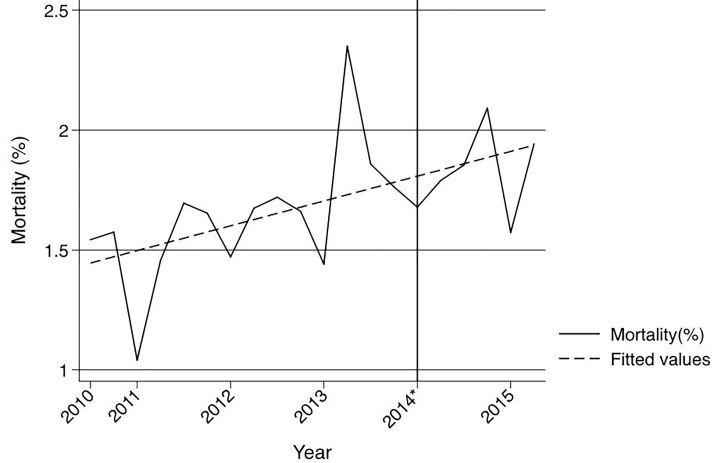
Crude mortality of Illinois trauma patients admitted through the emergency department from third quarter 2010 through second quarter 2015. *Indicates January 1, 2014, which marks the beginning of insurance expansion in Illinois.

There was also no significant change in the proportion of Medicare patients (0.3% 95% CI −0.81–0.18). There was a slight increase in overall crude mortality after the implementation of the ACA with an increase of 0.18% (95% CI 0.00, 0.04) (Figure 3).

In our adjusted models, we found that the uninsured died at a higher rate when compared to privately insured patients after adjusting for age, sex, race, low-income zip code, mechanism of injury, shock, TMPM score, Charlson Comorbidity Index, and hospital trauma volume with an IRR=1.24 (95% CI, 1.08–1.43) (Supplement). There was no significant change in adjusted trauma mortality comparing the post-ACA population with the pre-ACA population with an IRR=1.05 (95% CI 0.93–1.17) (Table 2).

**Figure 4:**
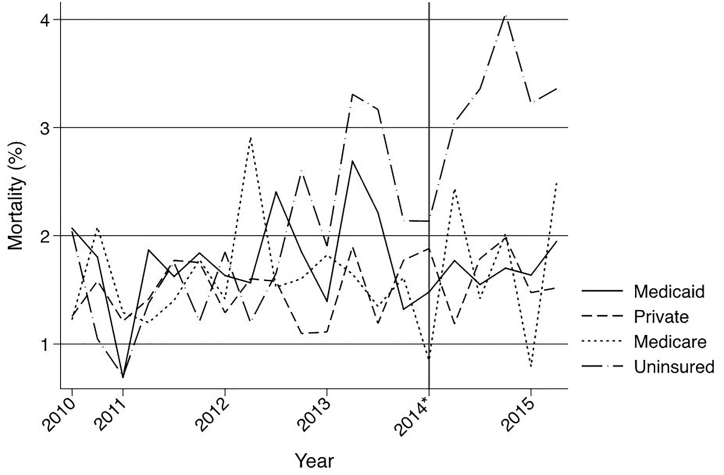
Crude mortality of Illinois trauma patients admitted through the emergency department from third quarter 2010 through second quarter 2015 by insurance type. *Indicates January 1, 2014, which marks the beginning of insurance expansion in Illinois.

However, among patients who remained uninsured after the implementation of the ACA crude mortality increased by 1.20% (95% CI 0.53, 1.88) (Figure 4), and adjusted mortality increased as well with an IRR=1.46 (95% CI, 1.14–1.88) (Table 3).

## DISCUSSION

Our results demonstrate that the patients who remained uninsured after ACA implementation in Illinois had a disproportionately high mortality after trauma. While the ACA-related insurance expansion significantly decreased the number of uninsured adults in Illinois, we did not find a significant decrease in overall trauma mortality after the expansion.

Our data support prior studies that have shown that being uninsured is an independent predictor of mortality after trauma.^2^ It might be hypothesized that these differences in mortality are due to mechanisms of injury, which can lead to differential mortality. Penetrating trauma is associated with high mortality rates;^30^ however, recent studies have shown that even among patients with blunt injuries alone, uninsured patients die at a higher rate.^6^ Many additional factors have been shown to affect mortality of patients presenting with trauma.

The ACA has provided increased coverage for the uninsured in the form of Medicaid expansion for adults up to 138% of the federal poverty level and the Health Insurance Marketplace, which provides access to insurance plans for those who do not qualify for Medicaid.^31^ Our study demonstrates that this program effectively reduced the number of uninsured patients in Illinois, confirming findings from prior studies.^15^ We hypothesized that if insurance is truly an independent predictor of trauma mortality, then mortality rates should fall after ACA-related insurance expansion, bearing in mind that many of the individual improvements in health status associated with insurance coverage may not be fully realized until years after the initial intervention. However, if hospital and provider factors were the major drivers of insurance-related disparities in outcomes, then we would expect a decrease in trauma mortality after ACA.

Osler et al. found that healthcare reform in Massachusetts paradoxically increased overall trauma mortality.^14^ While there was no significant change in overall trauma mortality after ACA insurance expansion in our study, we found that those patients who remain uninsured appear to suffer significantly higher mortality after ACA implementation. We are not suggesting that the ACA is causing a rise in mortality among the remaining uninsured patients as might be inferred from Figure 4, but rather we believe the group that remains uninsured after ACA healthcare expansion is suffering an unusually high burden of trauma-related mortality. Scott et al. suggested that since young people who benefit from the dependent healthcare expansion are mostly children of insured adults there would be increased disparities between the newly insured and the uninsured.^32^ At least a proportion of the increased mortality in our remaining uninsured population may be attributable to this phenomenon. Unmeasured factors are likely contributing to this increased burden of mortality as well. The insurance-related disparities observed in this study may reflect patient factors, hospital or provider factors, or unknown or unmeasured confounders.^2^ Unmeasured host-level cofounders in this study that could contribute to the undue burden of mortality in this group include obesity,^33^ homelessness,^34^,^35^ and immigration status.^36^,^37^

Future studies should focus efforts on determining the factors that lead to the high burden of mortality among the remaining uninsured. Better understanding of the underlying reasons for this increased mortality may lead policymakers to enact policies to reduce the disparity between patients who are insured and those who remain uninsured.

## LIMITATIONS

This study was limited to Illinois, and its generalizability to the greater United States remains uncertain. Our study was also limited by its retrospective nature and can therefore only comment on association and not causation. Our source, COMPData, provides administrative claims data, which are known to suffer certain limitations.^38^,^39^ We were unable to assess for the impact of proposed mechanisms behind trauma disparities including host factors such as homelessness, obesity, and both prehospital and post-hospital care. While we were able to adjust for comorbidities in our analysis, we were unable to control for undiagnosed comorbidities, which may be a partial driver of differential mortality in our dataset.

It has been suggested that patients who survive longer are more likely to obtain insurance before discharge thus biasing the uninsured group towards the more severely injured and falsely elevating the mortality in this group, a phenomenon known as survivor treatment assignment bias (STAB).^40^ It is certainly feasible that some of the effects of insurance are attributable to STAB; however, we believe that this does not explain the entire association we have identified in this study. To address this potential bias we performed a post-hoc sensitivity analysis restricted to patients who did not die within the first day of hospitalization; the IRR for death among uninsured patients post-ACA was 1.52 with p=0.03 and therefore did not significantly change our major findings. Finally, many of the proposed mechanisms for higher mortality among uninsured patients after trauma are related to the overall health status of individuals, and the effects of preventative care and health maintenance may take years to reach full effect.

## CONCLUSION

Uninsured trauma patients die at higher rates in Illinois. Even after the ACA drastically reduced the number of uninsured patients in Illinois, trauma mortality has not fallen. The group that remains uninsured after ACA implementation appears to be suffering a higher burden of mortality. Researchers and policymakers should focus on this vulnerable group to reduce or eliminate these ongoing disparities.

## Supplementary Information



## Figures and Tables

**Table 1:** Baseline characteristics of Illinois trauma patients admitted through the emergency department from third quarter 2010 through second quarter 2015.

Variable	Category	Overall	Pre-ACA	Post-ACA
Patient ID (total)		87,001	62,018	24,983
Mortality (%)		1.69	1.64	1.83
Insurance (%)	Private	48.48	49.17	46.75
	Medicaid	19.38	15.69	28.55
	Medicare	13.00	12.90	13.22
	Uninsured	19.14	22.23	11.48
Age (%)	18–25	15.72	16.11	14.76
	26–33	12.55	12.77	11.99
	34–45	18.23	18.55	17.42
	46–55	25.26	25.42	24.85
	56–64	28.25	27.15	30.97
Male (%)		65.39	65.40	65.38
Race (%)	Black	21.50	21.28	22.05
	White	58.69	59.93	55.60
	Latino	12.63	12.54	12.86
	Other	7.18	6.24	9.49
Low-income (%)	(<$35,000)	14.09	13.88	14.61
Mechanism	GSW-SI	0.17	0.16	0.18
	GSW	5.84	5.52	6.63
	MVC	21.17	21.97	19.19
	Cut/Pierce	4.95	5.08	4.62
	Blunt (no MVC)	17.61	17.93	16.81
	Falls	50.26	49.33	52.57
Shock (%)		1.01	0.94	1.18
Comorbidities	Not ill	72.37	73.69	69.09
	Moderately ill	22.07	21.22	24.18
	Severely ill	5.56	5.08	6.73
TMPM (median %)		1.33	1.33	1.33
TRVOL	<110.2	25.43	25.47	25.33
	110.3–248.4	24.63	24.32	25.41
	284.5–568.8	27.89	27.31	29.33
	568.9+	22.05	22.91	19.93

	*N*	87,001		

*ACA*, Affordable Care Act; *GSW*, gunshot wound; *SI-GSW*, self-inflicted GSW; *MVC*, motor vehicle collision; *TMPM*, probability of death as provided by the Trauma Mortality Prediction Model; *TRVOL*, trauma visit volume quartiles.

Volume quartiles indicate average, yearly, inpatient, hospital trauma volume over the study period.

**Table 2:** Regression model evaluating effects of post-ACA* period and covariates on inpatient trauma mortality in Illinois.

Variable	Category	IRR	95% CI	p
Post-ACA		1.05	0.93 – 1.17	0.43
Age (18–24)	26–33	1.04	0.86 – 1.25	0.71
	36–45	1.00	0.82 – 1.21	0.97
	46–55	1.51	1.26 – 1.80	0.00
	55–64	1.53	1.27 – 1.84	0.00
Male		1.34	1.18 – 1.51	0.00
Race (White)	Other	1.21	1.00 – 1.47	0.05
	Black	0.95	0.82 – 1.10	0.47
Mechanism (Falls)	Latino	0.83	0.70 – 0.99	0.04
	Self-inflicted GSW	3.20	2.31 – 4.42	0.00
	GSW	1.95	1.57 – 2.42	0.00
	MVC	1.24	1.07 – 1.43	0.00
	Cut/Pierce	0.51	0.35 – 0.74	0.00
	Blunt (no MVC)	0.63	0.51 – 0.77	0.00
Shock		3.21	2.71 – 3.79	0.00
Low-Income	(68864+)	0.97	0.83 – 1.13	0.66
Comorbidities (Not ill)	Moderately Ill	1.93	1.71 – 2.19	0.00
	Severely Ill	4.52	3.82 – 5.34	0.00
	TMPM	177.66	140.55 – 224.57	0.00
TRVOL (Quartile 1)	Quartile 2	1.44	1.18 – 1.74	0.00
	Quartile 3	2.09	1.76 – 2.50	0.00
	Quartile 4	1.99	1.65 – 2.41	0.00
Quarter (Quartile 1)	Quarter 2	1.23	1.05 – 1.43	0.01
	Quarter 3	1.22	1.05 – 1.42	0.01
	Quarter 4	1.23	1.05 – 1.44	0.01

	*N*	87,001		

* *ACA*, Affordable Care Act; *IRR*, incidence rate ratio; *GSW*, gunshot wound; *MVC*, motor vehicle collision; *TMPM*, probability of death as provided by the Trauma Mortality Prediction Model; *TRVOL*, trauma visit volume quartiles.

Volume quartiles indicate average yearly inpatient hospital trauma volume over the study period. Reference groups are provided in parentheses.

**Table 3:** Regression model evaluating effects of post-ACA* period and covariates on uninsured inpatient trauma mortality in Illinois.

Variable	Category	IRR	95% CI	p
Post-ACA		1.46	1.14 – 1.88	0.00
Age (18–24)	26–33	1.01	0.74 – 1.37	0.97
	36–45	0.85	0.59 – 1.22	0.38
	46–55	1.67	1.19 – 2.34	0.00
	55–64	1.79	1.20 – 2.69	0.00
Male		1.20	0.86 – 1.66	0.29
Race (White)	Other	1.53	1.05 – 2.24	0.03
	Black	1.06	0.78 – 1.45	0.70
Mechanism (Falls)	Latino	1.00	0.72 – 1.39	0.98
	Self-inflicted GSW	3.89	2.08 – 7.26	0.00
	GSW	2.31	1.55 – 3.44	0.00
	MVC	1.74	1.25 – 2.41	0.00
	Cut/Pierce	0.72	0.41 – 1.26	0.25
	Blunt (no MVC)	0.61	0.39 – 0.95	0.03
Shock		2.83	2.04 – 3.93	0.00
Low-Income	(68864+)	0.92	0.70 – 1.22	0.56
Comorbidities (Not ill)	Moderately Ill	1.91	1.46 – 2.51	0.00
	Severely Ill	2.81	1.50 – 5.26	0.00
	TMPM	157.11	101.66 – 242.81	0.00
TRVOL (Quartile 1)	Quartile 2	1.18	0.72 – 1.92	0.51
	Quartile 3	1.70	1.11 – 2.61	0.02
	Quartile 4	1.50	0.96 – 2.34	0.07
Quarter (Quartile 1)	Quarter 2	1.31	0.94 – 1.82	0.11
	Quarter 3	1.43	1.04 – 1.97	0.03
	Quarter 4	1.37	0.97 – 1.93	0.07

	*N*	16,655		

* *ACA*, Affordable Care Act; *IRR*, incidence rate ratio; *GSW*, gunshot wound; *SI-GSW*, self-inflicted GSW; *MVC*, motor vehicle collision; *TMPM*, probability of death as provided by the Trauma Mortality Prediction Model; *TRVOL*, trauma visit volume quartiles.

Volume quartiles indicate average yearly inpatient hospital trauma volume over the study period. Reference groups are provided in parentheses.
