# Supplementary material for: Inpatient Trauma Mortality after Implementation of the Affordable Care Act in Illinois
Source: West J Emerg Med. 2018 Feb 19;19(2):301–10. doi: 10.5811/westjem.2017.10.34949 (PMC5851503; doi:10.5811/westjem.2017.10.34949)
Supplement: Supplementary file 1 [file wjem-19-301-s001.docx]

**Supplement**. Regression model evaluating effects of insurance and covariates on inpatient trauma mortality in Illinois.

|  | Category | IRR | 95% CI | p |
| --- | --- | --- | --- | --- |
| Insurance | Medicaid | 0.94 | 0.81 - 1.09 | 0.40 |
| (Private) | Medicare | 1.01 | 0.85 - 1.20 | 0.93 |
|  | **Uninsured** | **1.24** | **1.08 - 1.42** | **0.00** |
| Age | 26-33 | 1.03 | 0.85 - 1.24 | 0.77 |
| (18-24) | 36-45 | 0.99 | 0.81 - 1.20 | 0.90 |
|  | 46-55 | 1.52 | 1.26 - 1.82 | 0.00 |
|  | 55-64 | 1.56 | 1.29 - 1.88 | 0.00 |
| Male |  | 1.31 | 1.15 - 1.48 | 0.00 |
| Race (White) | Other | 1.22 | 1.01 - 1.47 | 0.04 |
|  | Black | 0.94 | 0.81 - 1.09 | 0.42 |
|  | Latino | 0.82 | 0.68 - 0.98 | 0.03 |
| Mechanism  (Falls) | SI-GSW | 3.19 | 2.31 - 4.41 | 0.00 |
|  | GSW | 1.93 | 1.56 - 2.40 | 0.00 |
|  | MVC | 1.24 | 1.07 - 1.44 | 0.01 |
|  | Cut/Pierce | 0.50 | 0.35 - 0.72 | 0.00 |
|  | Blunt (no MVC) | 0.62 | 0.51 - 0.76 | 0.00 |
| Shock |  | 3.19 | 2.70 - 3.78 | 0.00 |
| Low-income | (<$35,000) | 0.97 | 0.83 - 1.13 | 0.68 |
| Comorbidities | Moderately Ill | 1.96 | 1.73 - 2.22 | 0.00 |
| (Not ill) | Severely Ill | 4.63 | 3.89 - 5.49 | 0.00 |
| TMPM |  | 177.62 | 140.44 - 224.63 | 0.00 |
| TRVOL | Quartile 2 | 1.43 | 1.18 - 1.73 | 0.00 |
| (Quartile 1) | Quartile 3 | 2.08 | 1.74 - 2.48 | 0.00 |
|  | Quartile 4 | 1.97 | 1.63 - 2.39 | 0.00 |
| (1^st^ Quarter) | 2^nd^ Quarter | 1.23 | 1.06 - 1.44 | 0.01 |
|  | 3^rd^ Quarter | 1.21 | 1.04 - 1.40 | 0.02 |
|  | 4^th^ Quarter | 1.23 | 1.05 - 1.43 | 0.01 |
|  | *N* |  | 87,001 |  |

IRR=Incidence Rate Ratio; GSW=gunshot wound; SI-GSW=Self-inflicted GSW; MVC=motor vehicle collision; TMPM=probability of death as provided by the Trauma Mortality Prediction Model; TRVOL=trauma visit volume quartiles. Volume quartiles indicate average yearly inpatient hospital trauma volume over the study period. Reference groups are provided in parentheses.
